# Postoperative Drain Site Seeding to the Abdominal Wall of Sigmoid Adenocarcinoma: A Case Report and Literature Review

**DOI:** 10.7759/cureus.26118

**Published:** 2022-06-20

**Authors:** Edgar Theodore T Polintan, Francis Jestin Aquino, Wilfredo Liangco, Rommel Lojo

**Affiliations:** 1 Faculty of Medicine and Surgery, University of Santo Tomas, Manila, PHL; 2 Department of Internal Medicine, Mary Mediatrix Medical Center, Lipa, PHL; 3 Department of Surgery, Mary Mediatrix Medical Center, Lipa, PHL

**Keywords:** metastasis, drain site, abdominal wall tumor, sigmoid adenocarcinoma, colorectal cancer

## Abstract

Colorectal carcinoma (CRC) is a very common cancer found worldwide. When metastasizing, it would often seed the liver via traveling through the portal circulation; however, locoregional metastasis is also possible. Abdominal wall seeding postoperatively has been described to happen rarely in those who underwent definitive surgery for CRC. Currently, five case reports are in publication describing this phenomenon.

Here, we present a case of a drain site abdominal wall tumor recurrence after definitive surgery with curative intent of a sigmoid adenocarcinoma. Those with higher tumor-node-metastasis (TNM) staging and a primary site at the sigmoid colon were found to be at a higher risk for recurrence. Despite this, abdominal wall recurrence of CRC is exceptionally rare, with less than 1% of those with locoregional recurrence presenting at the incision site or trocar site placement.

Because of the rarity of this complication, few studies are available that detail the management of abdominal wall recurrence of CRC. Further studies on this subject are currently warranted.

## Introduction

Colorectal carcinoma (CRC) is a very common cancer in the Philippines. It accounts for 7.4% of all cancers in the country and is ranked as the third most commonly diagnosed primary cancer, with 11,315 newly reported cases diagnosed in 2020. It is also the fourth leading cause of cancer mortality in the country, causing 6.6% of all cancer-related deaths [1].

In the United States, a total of 106,180 new cases are expected in 2022, with a probability of developing invasive CRC at 4.2% and 4.0% for males and females, respectively. The estimated number of deaths due to CRC in 2022 is projected to be 52,580 in the United States alone [2].

The mainstay of treatment for CRC is surgical resection for Stages I to III cancers or for otherwise locally constrained diseases. However locoregional recurrence is noted to occur in approximately 11.5-12.3% of all cases post-resection [3-4]. A higher T- or N- grading and primary sites in the right colonic flexure or sigmoid colon were found to be independent risk factors that predispose to locoregional recurrence [4].

Despite this, drain site seeding of CRC to the abdominal wall is a very rare occurrence. There are currently only five case reports in publication on abdominal wall tumors secondary to drain site seeding of CRC from 1999 to 2018 upon literature review [5-9]. This displays a lack of data on this specific complication of CRC.

In light of this, we describe our own experience with a case of abdominal wall seeding of sigmoid adenocarcinoma secondary to drain site placement after curative resection.

## Case presentation

**Figure 1:**
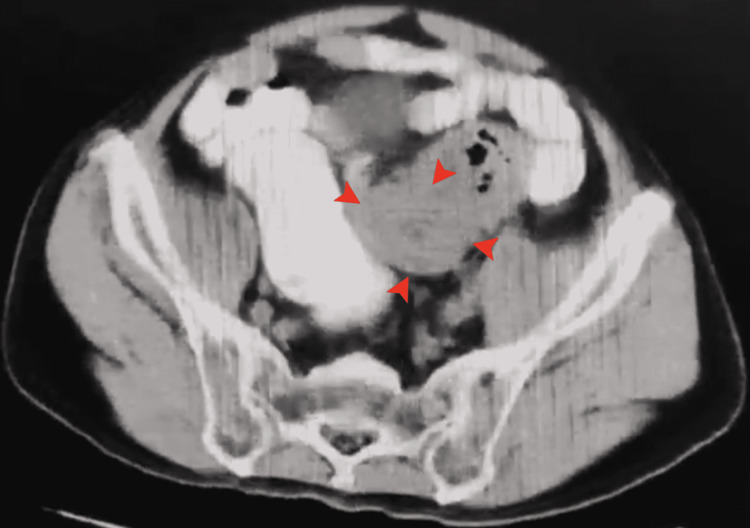
Transverse plain CT scan: arrows point to a concentric obstructive mass measuring 7-8 cm in the sigmoid colon

A 70-year-old Southeast Asian male would come to our institution due to a nine-month history of hematochezia, which was associated with a decrease in stool caliber. He would undergo colonoscopy, which showed the presence of a friable, fungating, colonic mass 45 cm from the anal verge. Upon biopsy, histopathology would confirm malignancy with sigmoid adenocarcinoma. This was followed by a comprehensive work-up, revealing a concentric bowel mass measuring approximately 8 x 3 cm with the involvement of 5 mm paracolic nodes on a whole abdominal CT scan leading to a tumor-node-metastasis (TNM) staging of stage IIIC (T3N1M0) preoperatively (Figure 1).

**Figure 2:**
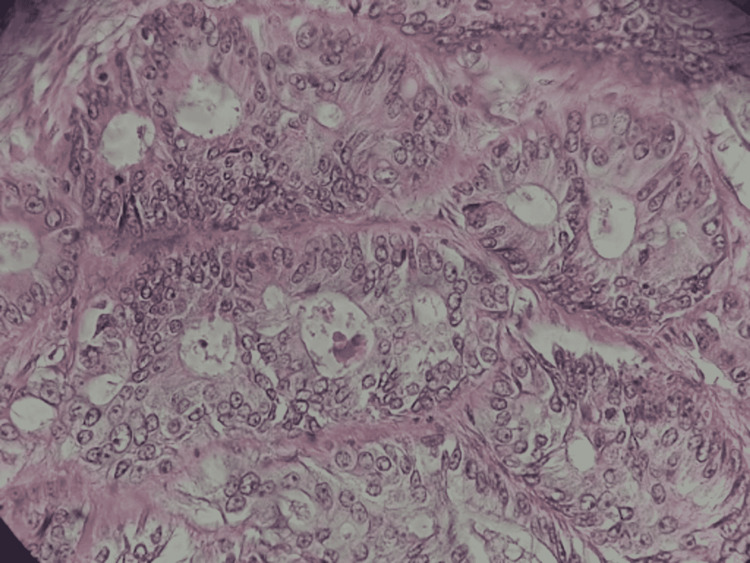
Primary site histopathology: well-differentiated adenocarcinoma showing multiple overlapping glandular formations with desmoplastic stroma

A left hemicolectomy with end-to-end anastomosis and Jackson Pratt drain placement would be done. Intraoperatively, it was found that the 8 x 6 cm mass was adherent to the urinary bladder wall, which necessitated a cystorrhaphy procedure. Adherence to the bladder wall upstaged the patient’s TNM staging to stage IIIC (T4bN1M0). Postoperative histopathology would report a well-differentiated adenocarcinoma with infiltration beyond the muscularis up to the pericolic soft tissue and subserosa with involvement of two out of 11 pericolic lymph nodes (Figure 2). Negative tumor margins were also achieved in histopathology. Postoperatively, he would be monitored with surveillance colonoscopy and started on six cycles of adjuvant chemotherapy with capecitabine 1000 mg/m^2^ twice on Days 1 to 14 as well as oxaliplatin 130 mg/m^2^ on Day 1 of a 21-day cycle.

One year postoperatively, the patient would present with an ulcerative lesion on the previous drain site at the right lower abdominal quadrant, which was initially diagnosed as a chronic wound infection. Specimens from a wound debridement revealed the presence of malignant tissue with a well-differentiated adenocarcinomatous histology, which was accompanied by acute suppurative inflammation. A wide excision was offered for the noted abdominal wall recurrence but the patient elected to undergo repeat chemotherapy alone. He was subsequently given a modified chemotherapy regimen with a total of four cycles of irinotecan 200 mg/m^2^ on Day 1 and capecitabine 800m/m^2^ twice a day on Days 1 to 14 of a 21-day cycle due to disease progression, and tumor surveillance was resumed.

**Figure 3:**
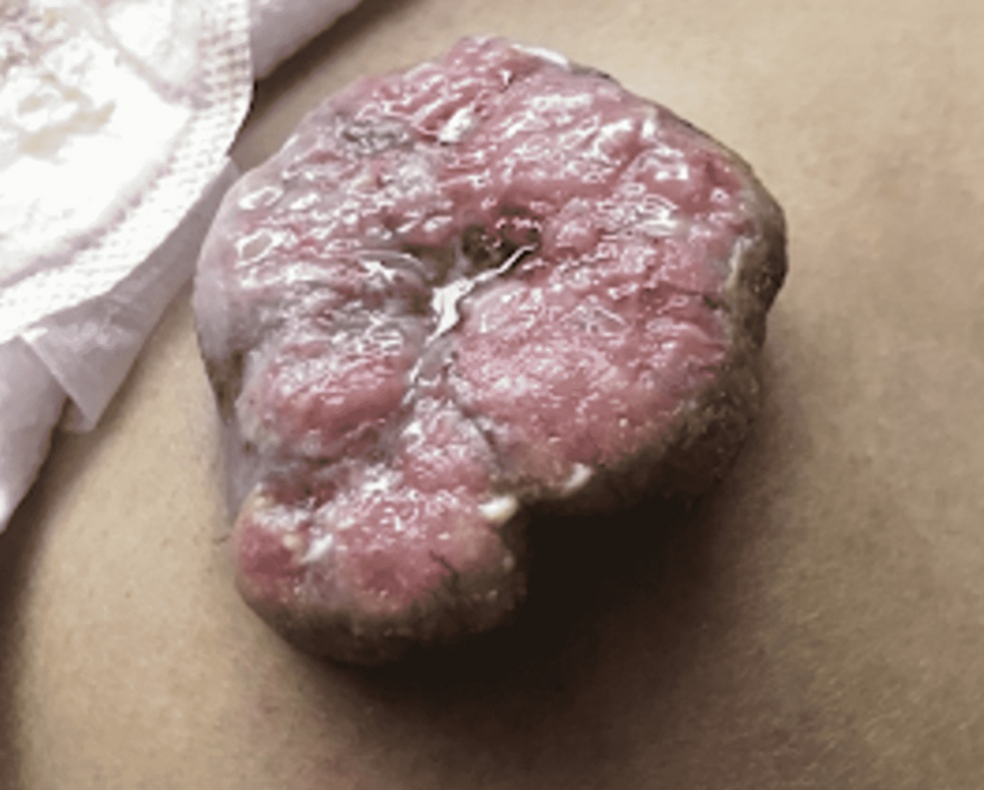
Gross appearance of the anterior abdominal wall mass at the previous drain site

**Figure 4:**
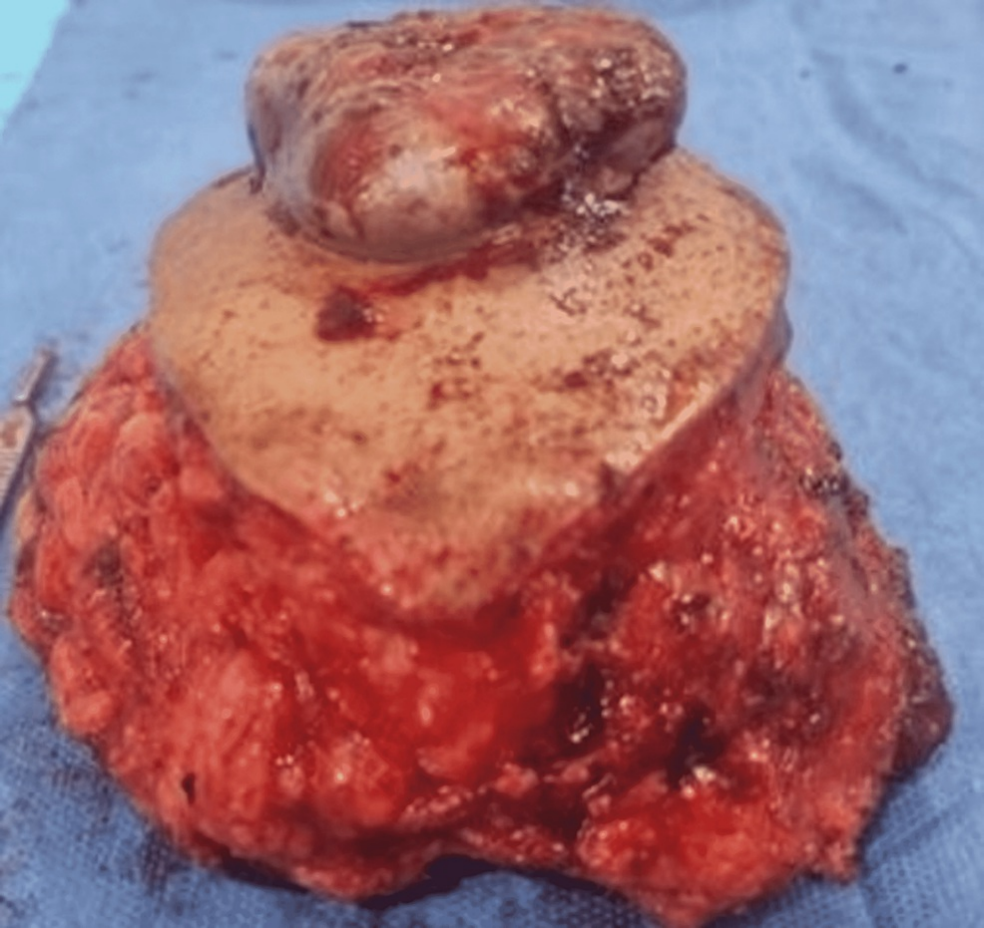
Postoperative appearance of the abdominal wall mass

**Figure 5:**
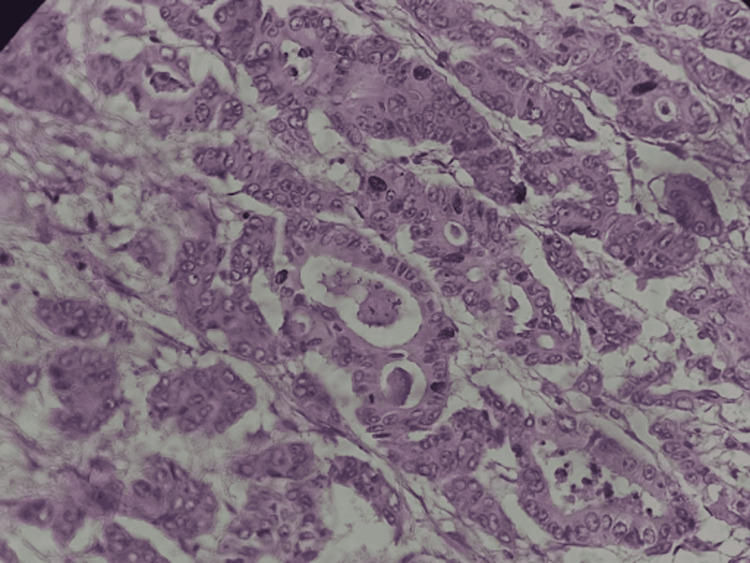
Abdominal wall mass histopathology: well-differentiated adenocarcinoma with multiple overlapping glandular formations similar to the primary carcinoma

Two years postoperatively, the previous drain site would develop a 10 x 8 x 6 cm fungating mass on the right lower abdominal wall (Figure 3 and Figure 4). Once the patient was found to be negative for distant metastasis on CT scan, an exploratory laparotomy with wide excision of the tumor was done. Histopathology exhibited the presence of adenocarcinoma with the same histologic characteristic as the primary tumor, favoring recurrence, with surgical margins negative for metaplasia (Figure 5). The patient would eventually be discharged and present well on further follow-ups.

## Discussion

The development of post-procedural tumor seeding is hypothesized to be due to micrometastasis from malignant cell exfoliation of the primary tumor site. It is a very rare complication that may occur following any medical procedure that involves the manipulation of a neoplasm. However, tumor seeding leading to cutaneous recurrence of CRC is a far rarer occurrence compared to other sites of loco-regional recurrence.

A single-center retrospective study on Chinese patients by Hu et al. showed that only 0.81% of CRC metastasize to the skin [10]^ ^while the study by Lookingbill et al. revealed a 4.4% rate of metastasis from CRC to the skin in Caucasian patients [11]. The study by Hu et al. did not make a distinction between postprocedural metastasis from distant metastasis while the study by Lookingbill et al. implies that a majority of CRC cutaneous metastasis may be related to postprocedural neoplastic colonization due to 11 out of 18 cutaneous metastasis appearing at the wound incision site. Both studies are limited in their applicability to other ethnicities due to the homogeneity of their study population.

A five-year incidence of locoregional recurrence was estimated to be 11.5% [4]; however, it was found that only 0.6% were at risk of trocar placement site metastasis in those undergoing laparoscopic colectomy [12] while a similar risk of 0.6% for incisional recurrence was seen in those undergoing open colectomy [13], indicating that a laparoscopic or open approach may not significantly affect the presence of an abdominal wall or cutaneous metastasis.

Studies on the occurrence of drain site metastasis itself are not well-established, as there is a paucity of relevant literature describing the risk of recurrence through postoperative drain site seeding. Regardless, due to the very low incidence of abdominal wall recurrence, there is no specific management recommendation currently available. In our patient, the cutaneous recurrence was managed similarly to a malignant cutaneous carcinoma with wide excision of the tumor. Wide excision of an abdominal wall recurrence of CRC was found to be associated with a survival rate of 87.7% with a recurrence rate of 13.3% as stated in a retrospective study of 15 patients followed for four months to five years [14].

## Conclusions

Abdominal wall recurrence is a rare complication of primary colorectal carcinomas. This has led to a scarcity of information regarding the management of CRC patients presenting with an abdominal wall recurrence. Additionally, a majority of the available literature on this subject is more than 10 years from its publication, which indicates a need for more contemporary data. Hence, we recommend more studies on the efficacy of the management and prognosis in CRC with isolated abdominal wall recurrence.
